# Cost–Consequence Analysis of Semaglutide vs. Liraglutide for Managing Obese Prediabetic and Diabetic Patients in Saudi Arabia: A Single-Center Study

**DOI:** 10.3390/healthcare13141755

**Published:** 2025-07-20

**Authors:** Najla Bawazeer, Seham Bin Ganzal, Huda F. Al-Hasinah, Yazed Alruthia

**Affiliations:** 1 Department of Pharmacy, Prince Sultan Military Medical City, Riyadh 11159, Saudi Arabia; nbawazeer@psmmc.med.sa (N.B.); salganzal@psmmc.med.sa (S.B.G.); haldossari@psmmc.med.sa (H.F.A.-H.); 2 Department of Clinical Pharmacy, College of Pharmacy, King Saud University, Riyadh 11451, Saudi Arabia

**Keywords:** diabetes, prediabetes, Saudi Arabia, Semaglutide, Liraglutide, obesity, cost-effectiveness

## Abstract

**Background**: Semaglutide and Liraglutide are medications in the Glucagon-like peptide-1 agonists (GLP-1 RAs) class used to manage type 2 diabetes mellitus and obesity in Saudi Arabia. Although the 1.0 mg once weekly dosage of Semaglutide does not have a labeled indication for the management of obesity, many believe that this dosage is more effective than the 3.0 mg once daily Liraglutide dosage for the management of both diabetes and obesity. **Objective**: To compare the effectiveness of the dosage of 1.0 mg of Semaglutide administered once weekly versus 3.0 mg of Liraglutide administered once daily in controlling HbA1c levels, promoting weight loss, and evaluating their financial implications among obese patients in Saudi Arabia using real-world data. **Methods**: A retrospective review of Electronic Medical Records (EMRs) from January 2021 to June 2024 was conducted on patients prescribed Semaglutide or Liraglutide for at least 12 months. Exclusion criteria included pre-existing severe conditions (e.g., cardiovascular disease, stroke, or cancer) and missing baseline data. The primary outcomes assessed were changes in HbA1c, weight, and direct medical costs. **Results**: Two hundred patients (100 patients on the 1.0 mg once weekly dose of Semaglutide and 100 patients on the 3.0 mg once daily dose of Liraglutide) of those randomly selected from the EMRs met the inclusion criteria and were included in the analysis. Of the 200 eligible patients (65.5% female, mean age 48.54 years), weight loss was greater with Semaglutide (−8.09 kg) than Liraglutide (−5.884 kg). HbA1c reduction was also greater with Semaglutide (−1.073%) than Liraglutide (−0.298%). The use of Semaglutide resulted in lower costs of USD −1264.76 (95% CI: −1826.82 to 33.76) and greater reductions in weight of −2.22 KG (95% CI: −7.68 to −2.784), as well as lower costs of USD −1264.76 (95% CI: (−2368.16 to −239.686) and greater reductions in HbA1c of −0.77% (95% CI: −0.923 to −0.0971) in more than 95% of the cost effectiveness bootstrap distributions. **Conclusions**: Semaglutide 1.0 mg weekly seems to be more effective and cost-saving in managing prediabetes, diabetes, and obesity compared to Liraglutide 3.0 mg daily. Future studies should examine these findings using a more representative sample and a robust study design.

## 1. Introduction

Obesity is defined as “abnormal or excessive fat accumulation that presents a health risk” characterized by a body mass index (BMI) of 30 kg/m^2^ or higher [1]. It is a multisystem disease linked to an increased risk of non-communicable diseases (NCDs) like cardiovascular disease, diabetes, cancer, and chronic respiratory disease. Since 1990, adult obesity has more than doubled worldwide, with 2.5 billion overweight adults in 2022, including 890 million classified as obese [2,3]. Projected figures suggest that by 2035, over 4 billion people, or 50% of the world’s population, may be affected [4]. In Saudi Arabia, the 2019 World Health Survey reported an adult obesity rate of 20.2% and overweight at 38.2%, with higher rates in women [5]. By 2035, the prevalence of adult obesity is expected to reach 57% [4]. Obesity significantly contributes to various health issues, including type 2 diabetes and coronary heart disease [6]. It is responsible for around 43% of type 2 diabetes cases globally, resulting in 4 million deaths, with major complications anticipated to cost around USD 1.2 trillion by 2025 globally [7].

The prevalence of type 2 diabetes will continue to rise unless we make it an urgent priority to address obesity, which is a significant contributing factor. There is growing concern regarding prediabetic individuals, as they often exhibit a high prevalence of traditional cardiovascular disease (CVD) risk factors, including dyslipidemia, obesity, and hypertension [8]. These individuals are at an increased risk for diabetes progression, cardiovascular disease, chronic kidney disease, retinopathy, and other complications related to diabetes. Currently, the global prevalence of prediabetes among adults is approximately 7.3%, with about 70% of prediabetics likely to progress to overt type 2 diabetes mellitus (T2DM) [8,9].

The estimated annual direct medical cost of obesity is approximately USD 3.8 billion, accounting for 4.3% of health expenditures, and is projected to exceed 7% of total annual health expenditures between 2020 and 2050 [10]. Furthermore, absenteeism attributed to obesity may reduce gross domestic product (GDP) estimates by 1.42% annually [7]. Therefore, addressing obesity is a pressing public health concern that places a significant burden on the healthcare system.

Various treatment approaches are available for managing obesity, including pharmacological and non-pharmacological therapies [11]. Pharmacological therapies are categorized into oral and injectable medications, each with its distinct classes. Orlistat, an irreversible pancreatic lipase inhibitor, is associated with gastrointestinal side effects and is recommended for use alongside a low-fat diet. Other agents, such as Lorcaserin, work as a serotonin agonist to centrally suppress appetite, while phentermine/topiramate is a sympathomimetic agent that promotes weight loss through its cardiovascular effects; it should be used cautiously in patients with hypertension [10,11]. The guidelines developed by the American College of Cardiology (ACC), the American Heart Association (AHA), and The Obesity Society (TOS) recommend antiobesity medications in conjunction with lifestyle modifications for individuals with a BMI of at least 27 kg/m^2^ who have at least one weight-related complication (such as hypertension, type 2 diabetes mellitus, or dyslipidemia) or a BMI of at least 30 kg/m^2^. For the prolonged management of obesity, GLP-1 receptor agonists (GLP-1 RAs), such as Semaglutide (Wegovy^®^) or Liraglutide (Saxenda^®^), in addition to Orlistat, phentermine/topiramate, and naltrexone/bupropion, should be prescribed [12]. The selection of medication should be personalized based on clinical weight loss objectives, weight-related conditions, and drug warnings [12]. Semaglutide and Liraglutide are GLP-1 receptor agonists approved for treating type 2 diabetes and obesity at higher doses. They work by reducing appetite, slowing gastric emptying, and lowering blood glucose levels. In addition to helping to lower blood sugar levels and assisting with weight management, these medications can also contribute to a reduction in fatty liver, support heart and kidney health, lower cardiovascular risks, and slow the progression of diabetic nephropathy [13]. Recent studies suggest that Semaglutide may have a more pronounced effect on reducing major adverse cardiovascular events (MACE) and may lead to greater weight loss compared to Liraglutide [13]. Nausea, vomiting, and constipation are common side effects associated with the use of Semaglutide and Liraglutide. While hypoglycemia is rarely seen with these medications, it can occur in patients who are also taking other antihyperglycemic agents [14,15]. Semaglutide and Liraglutide are considered first-line treatment options for managing obesity, particularly in individuals who are prediabetic or diabetic [13]. These medications play a crucial role in improving metabolic health and facilitating weight loss [12,16]. Despite their proven efficacy, the significant barriers to the use of GLP-1 RAs include patient access and high medication costs [17]; some studies have indicated that a Semaglutide 1.0 mg dose administered once weekly (QW) is the most cost-effective GLP-1RA among other GLP-1RAs, such as Dulaglutide, Exenatide, or Liraglutide, from the U.S. healthcare payer’s perspective using a threshold of USD 195,000 per Quality Adjusted Life Year (QALY) [18]. Additionally, the Semaglutide 1.0 mg dose administered once weekly (QW) was found to be less costly than Liraglutide, Dulaglutide, Exenatide, and Lixisenatide for the management of diabetes among a hypothetical cohort of diabetic patients in Saudi Arabia according to a budget impact model that estimated the cost of control per patient per year (CCPPPY) over a 5-year horizon (2021–2025) [17]. Currently, there has been no research investigating the cost-effectiveness of the Semaglutide 1.0 mg dose administered once weekly (QW) in comparison to other GLP-1 receptor agonists (GLP-1RAs) such as Liraglutide, specifically for treating obese diabetic and prediabetic patients in Saudi Arabia. The importance of conducting an economic evaluation of the Semaglutide 1.0 mg dose QW versus Liraglutide administered at 3 mg/day for obesity management is underscored by the fact that the former dosage has not yet received approval for this indication, while its acquisition cost is lower than the approved obesity dosage of 2.4 mg QW. Furthermore, if the Semaglutide 1.0 mg QW demonstrates non-inferiority to the Liraglutide 3 mg/day dosing in managing diabetes, prediabetes, and obesity, it could lead to significant cost savings for healthcare payers. Therefore, the aims of this study are to examine the cost and clinical consequences associated with the use of Semaglutide versus Liraglutide for the management of obesity and elevated HbA1c levels among diabetic and prediabetic patients in Saudi Arabia.

## 2. Materials and Methods

### 2.1. Study Design and Population

A single-center, retrospective medical chart review was conducted at the outpatient obesity and endocrinology clinics of Prince Sultan Military Medical City (PSMMC). Data were extracted from electronic medical records (EMRs) covering the period from January 2021 to June 2024. The study included patients who met specific eligibility criteria: male and female individuals aged 18 years and older who were initiated on Semaglutide for the treatment of type 2 diabetes mellitus (T2DM) for a minimum of 12 months or on Liraglutide for obesity for at least 12 months. Additional criteria required that baseline HbA1c readings be 5.7% or higher for those using Liraglutide and 6.5% or greater for those on Semaglutide. Furthermore, patients needed to have a baseline body mass index (BMI) of 30 kg/m^2^ or above accompanied by at least one chronic health condition such as asthma, diabetes, hypertension, dyslipidemia, or sleep apnea.

Individuals who did not meet these eligibility criteria were excluded from the study. This exclusion applied to those with pre-existing cardiac disease or cancer, those who became pregnant after starting the medication, those who discontinued the medication due to intolerance, and those with incomplete baseline data for HbA1c, body weight, or BMI at the time of medication initiation. The EMRs of eligible patients were reviewed, and relevant variables were collected at three-month intervals throughout the study period.

### 2.2. Dosage Regimen for Semaglutide and Liraglutide

The treatment protocol for Semaglutide in patients diagnosed with type 2 diabetes mellitus (T2DM) and Liraglutide for the management of obesity at PSMMC adhered to a structured dose-escalation regimen. For Semaglutide, the initial dosage was set at 0.25 mg, administered subcutaneously on a weekly basis for the first month. Subsequently, the dosage was increased to 0.5 mg weekly during the second month. Based on the patient’s response to treatment, it was decided to further escalate the dosage to 1.0 mg weekly until the desired improvement in HbA1c levels was attained. In the case of Liraglutide, treatment commenced with an initial dose of 0.6 mg, which was administered subcutaneously once daily for one week. This dosage was then increased by 0.6 mg daily on a weekly basis, with a maximum allowable dosage of 3.0 mg per day. Following the initiation of treatment, patients were monitored closely over a period of 16 weeks, or for 12 weeks after reaching the maximum dosage, with an objective weight loss goal ranging from 5.0% to 7.0% of the baseline weight.

### 2.3. Study Perspective

This cost–consequence analysis was conducted from the perspective of a public healthcare payer, focusing exclusively on direct medical costs associated with patient care. The analysis covered a range of expense categories, including prescription medications, which represent the costs of drugs dispensed to patients; hospitalization, encompassing the expenses related to inpatient admissions; and the duration of stay in medical facilities, which can significantly influence overall costs. Furthermore, the analysis included laboratory tests, essential for diagnosing and monitoring health conditions, as well as imaging studies such as X-rays, MRIs, and CT scans that facilitated visual assessments of patient health. By concentrating on these direct medical costs, the analysis aimed to provide a comprehensive understanding of the financial implications of healthcare interventions from the viewpoint of a public healthcare payer in Saudi Arabia.

### 2.4. Study Variables

The clinical outcomes associated with the use of Semaglutide and Liraglutide were evaluated in terms of mean reductions in HbA1c (%) and weight (in kilograms). Additionally, the financial aspects of using Semaglutide compared to Liraglutide were assessed through the mean difference in annual direct medical costs between the two treatment groups. Sociodemographic factors, such as age and gender, alongside medical characteristics, including comorbidities, prescribed medications, duration of treatment with Semaglutide or Liraglutide, laboratory tests, imaging studies, hospitalizations, and emergency room visits, were also collected.

### 2.5. Statistical Analysis

In this study, we performed descriptive statistics to evaluate the baseline characteristics of patients in both treatment groups. Key metrics included the mean, standard deviation, median, interquartile range, frequencies, and percentages, which provided a clear overview of the demography and health profiles of the patients.

To enhance the comparability of the two groups—patients receiving Semaglutide versus those receiving Liraglutide—we utilized propensity score matching. This technique allowed us to align patients based on several confounding factors, including sociodemographic variables, duration of the disease, and various medical profiles that could influence treatment outcomes.

To further strengthen the robustness of our analysis, we used a bootstrapping technique consisting of 10,000 replications. This approach enabled us to accurately calculate 95% confidence intervals for both the clinical outcomes and the associated costs of treatment.

The analysis specifically concentrated on the direct medical costs incurred from the viewpoint of healthcare payers operating within public hospitals. We sourced detailed cost data concerning health services and medications from the Council of Cooperative Health Insurance (CCHI) database, which provides comprehensive pricing information for a wide range of healthcare services. All statistical evaluations and analyses were conducted using SAS^®^ version 9.4 software, developed by SAS Institute, located in Cary, NC, USA.

## 3. Results

### 3.1. Patients’ Characteristics

We identified a total of 7931 patients on Semaglutide (Ozempic^®^) from the outpatient endocrinology clinic and 1539 patients on Liraglutide (Saxenda^®^) from the outpatient obesity clinic. Then, the simple random sampling method was employed using Microsoft Excel Software^®^ to randomly select the 100 patients from each treatment arm based on the predefined eligibility criteria. We ultimately had a total of 200 patients.

Table 1 presents the baseline characteristics of the patients included in the study. A total of 200 medical charts were reviewed, showing that 131 patients (65.50%) were female, accounting for two-thirds of each treatment group. The mean age of patients receiving Liraglutide was significantly younger than that of those receiving Semaglutide (43.87 ± 10.87 years vs. 53.20 ± 11.60, *p*-value < 0.0001). In contrast, the mean baseline weight was slightly higher in the Liraglutide group compared to the Semaglutide group (99.70 ± 16.18 kg vs. 95.65 ± 18.75 kg, *p*-value = 0.1040). Both groups exhibited a mean BMI greater than 30 kg/m^2^, categorizing them as obese individuals; however, the mean BMI was marginally higher in the Liraglutide group.

All patients in the Semaglutide group were diagnosed with diabetes, resulting in a significantly higher mean baseline HbA1c when compared to the Liraglutide group (9.00 ± 1.83 vs. 5.92 ± 0.20, *p*-value < 0.0001). The follow-up duration for both groups was adequate, averaging 20.57 ± 6.88 months. The mean number of comorbidities was significantly lower in the Liraglutide group compared to the Semaglutide group (1.79 ± 1.52 vs. 2.28 ± 1.07, *p*-value 0.0092). However, the prevalence of cardiovascular disease (CVD) was notably higher in the Semaglutide group. At the same time, comorbidities such as osteoarthritis and chronic lower back pain were more common among patients taking Liraglutide. Additionally, the mean number of prescription medications was significantly greater in the Liraglutide group than in the Semaglutide group (5.96 ± 4.51 vs. 4.51 ± 1.72, *p*-value = 0.0032).

### 3.2. Weight and HbA1c Reductions

The analysis revealed a statistically significant mean difference in weight (measured in kilograms) when comparing baseline measurements to those taken after follow-up among patients in both the Liraglutide and Semaglutide treatment groups. Specifically, the mean weight change in the Liraglutide group was found to be −5.884 kg, with a 95% confidence interval (CI) ranging from −7.85 to −3.92, while the Semaglutide group observed a mean weight reduction of −8.09 kg, with a 95% CI between −9.99 to −6.20. The *p*-value for these findings was less than 0.0001, indicating a statistically significant difference. Similarly, there was a statistically significant difference in the mean HbA1c levels between baseline and follow-up for patients in the two treatment groups who received different treatments. In the Liraglutide group, the mean HbA1c change was −0.298%, with a 95% confidence interval ranging from −0.47 to −0.126 and a *p*-value of 0.0009, indicating a statistically significant improvement. In contrast, the Semaglutide group exhibited a more pronounced reduction in HbA1c, with a mean difference of −1.073% (95% CI: −1.45 to −0.69; *p*-value < 0.0001) (refer to Table 2).

### 3.3. Medical Costs

**Figure 1:**
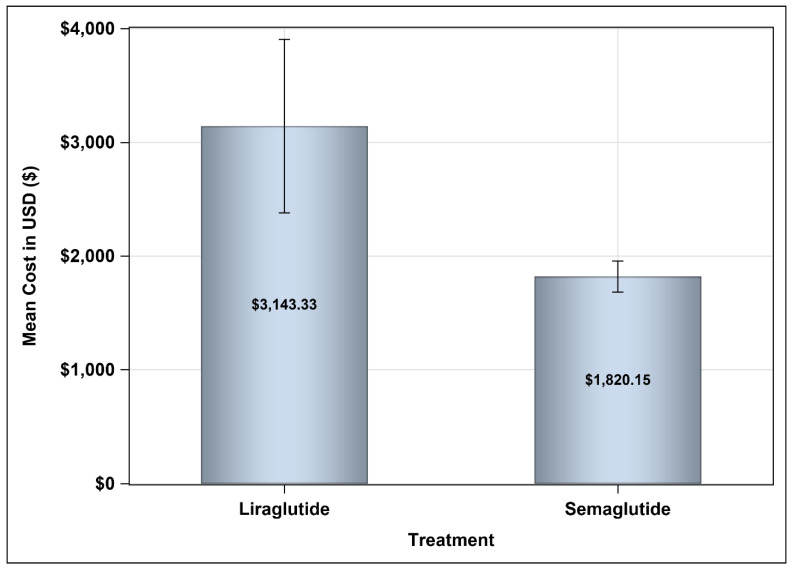
Mean annual pharmaceutical costs of Semaglutide and Liraglutide.

In analyzing the annual pharmaceutical costs associated with GLP-1 receptor agonists, Liraglutide was shown to exceed Semaglutide in terms of expenses, with reported average costs of USD 3143.33 compared to Semaglutide’s more affordable average of USD 1820.15, as illustrated in Figure 1. Additionally, the average annual direct medical cost for Semaglutide stood at USD 4523.16, accompanied by a standard deviation of USD 2176.04. In contrast, Liraglutide’s average annual direct medical costs reached USD 5787.92, featuring a larger standard deviation of USD 4606.97. This indicates a greater variability in total annual direct medical expenses for patients using Liraglutide. The mean difference of USD −1264.76 reflects a significant reduction in costs when opting for Semaglutide, with a 95% confidence interval ranging from −1826.82 to 33.76, as shown in Table 3.

**Figure 2:**
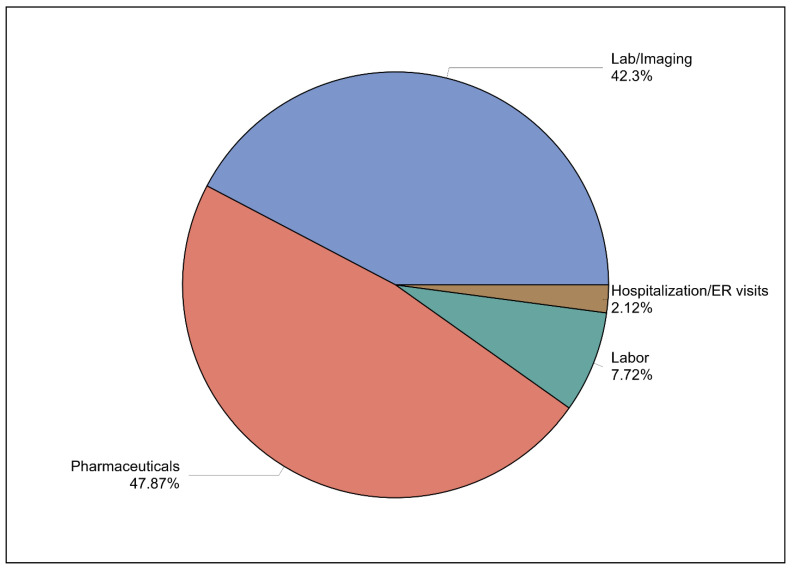
The breakdown of the annual direct medical costs for patients on GLP-1RAs.

A breakdown of the annual direct medical costs reveals that pharmaceuticals comprise the largest share at 47.87%, followed by laboratory and imaging expenses at 42.3%, labor costs at 7.72%, and hospitalizations and emergency room visit at 2.21%. This distribution of costs is visually represented in Figure 2.

### 3.4. Cost-Effectiveness Bootstrap Distributions

#### 3.4.1. Difference Between Semaglutide and Liraglutide in Weight Reduction

**Figure 3:**
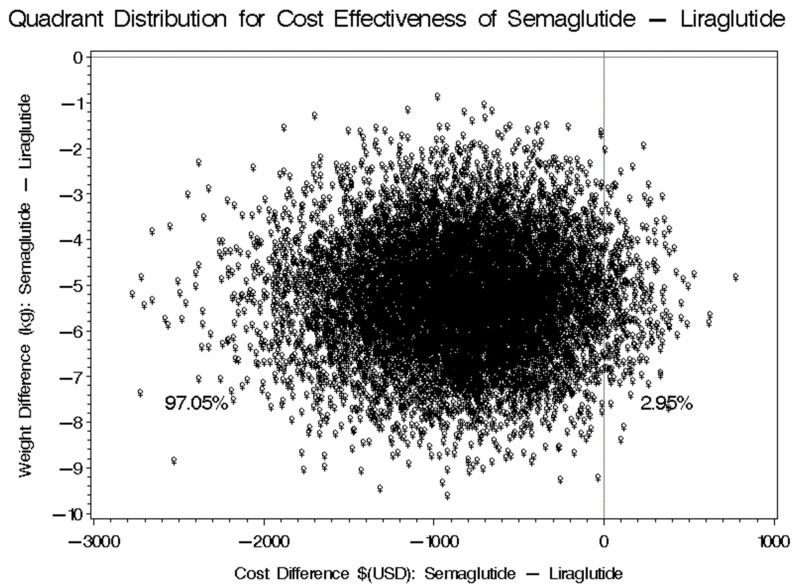
Cost-effectiveness bootstrap distributions for the difference in weight (kg).

Following a 12-month follow-up period, the average weight reduction demonstrated a significant difference between the two groups. Participants treated with Semaglutide experienced an average weight loss of −8.10 kg, with a standard deviation of ±9.55. In contrast, those on Liraglutide achieved an average weight loss of −5.88 kg, accompanied by a standard deviation of ±9.89. This resulted in a mean difference of −2.22 kg (95% CI: −7.68 to −2.784), indicating that Semaglutide was associated with a greater weight reduction compared to Liraglutide. The likelihood that Semaglutide will result in lower cost and greater weight reduction was 97.05%, as shown in Figure 3. However, there was a 2.95% chance estimated that Semaglutide will result in higher cost and greater weight reduction compared to Liraglutide.

Furthermore, the use of Semaglutide led to average cost savings of USD 1264.76 (95% CI: USD −1826.82 to 33.76), as shown in Table 3. Our analysis also controlled for six key variables that could potentially influence the results, including gender, baseline weight, metformin usage, the number of comorbidities, the total number of prescription medications being taken, and the duration of treatment with both Semaglutide and Liraglutide. Additionally, we controlled for the use of orally administered hypoglycemic agents to ensure a thorough understanding of the treatment effects.

#### 3.4.2. Difference Between Semaglutide and Liraglutide in HbA1c Reduction

**Figure 4:**
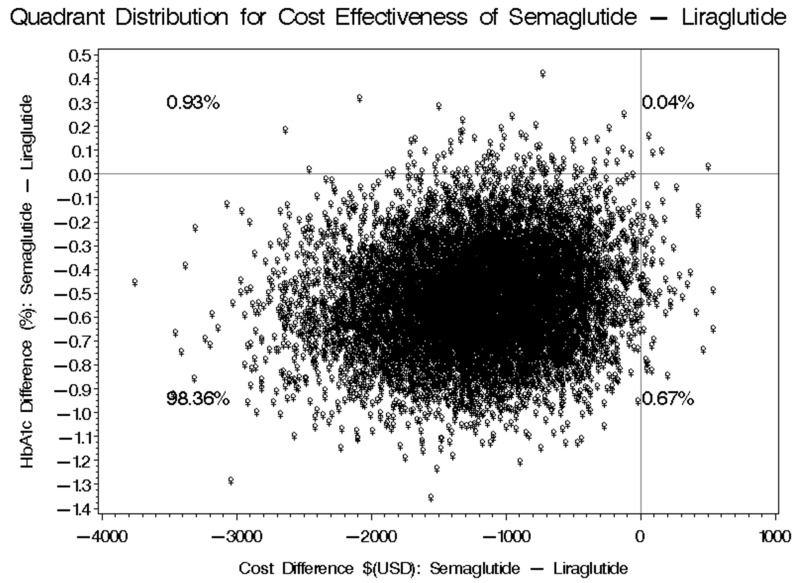
Cost-effectiveness bootstrap distributions for the difference in HbA1c.

Upon completing a 12-month follow-up period, the average reduction in HbA1c for patients on Liraglutide was −0.30% ± 0.87, whereas patients receiving Semaglutide experienced a greater reduction of −1.07% ± 1.91. This resulted in an overall mean difference of −0.77% in HbA1c levels, favoring Semaglutide, with a 95% confidence interval ranging from −0.923 to −0.0971 (refer to Table 4). Moreover, a bootstrap distribution analysis revealed that Semaglutide has a 98.36% probability of achieving a superior HbA1c reduction at a lower cost, a 0.67% chance of attaining a greater HbA1c reduction but at a higher cost, a 0.04% likelihood of achieving a lower reduction in HbA1c and higher costs, and a 0.93% probability of experiencing a lower HbA1c reduction with lower costs compared to Liraglutide, as illustrated in Figure 4. In our study, we controlled for several factors that could potentially affect the outcomes, adjusting for six specific variables: participant gender, baseline body weight, concurrent metformin use, total number of comorbidities, number of prescription medications, and the duration of treatment with both Semaglutide and Liraglutide. Additionally, we considered the use of other orally administered hypoglycemic agents to ensure a comprehensive evaluation of the treatment’s effects on HbA1c levels.

## 4. Discussion

The FDA has approved Semaglutide at a dosage of 1.0 mg as a treatment option for T2DM. This approval has resulted in Semaglutide capturing over 70% of the public-sector market share for GLP-1 receptor agonists in Saudi Arabia. In stark contrast, Liraglutide holds a market share of less than 1.5% [19]. Numerous studies have confirmed the effectiveness of Semaglutide within its pharmacological class [17,18]. However, our study is notable, as it represents the first comprehensive cost–consequence analysis that utilizes real-world data to assess the financial implications of Semaglutide 1.0 mg compared to Liraglutide 3.0 mg, particularly in terms of their impact on weight reduction and HbA1c levels. According to the American Diabetes Association (ADA) guidelines, a modest weight loss of 5 to 7% can lead to significant improvements in glycemic control for individuals who have diabetes, as well as decrease the risks of developing diabetes in those at risk [20]. Moreover, a sustained weight loss exceeding 10% may offer even greater metabolic benefits [21]. Our study emphasizes the importance of considering glycemic-lowering agents while selecting antiobesity medications (AOMs). Prioritizing the management of obesity as a distinct health condition is crucial, as it plays a pivotal role in postponing the transition from prediabetes to full-blown diabetes [22].

Our findings indicate that treatment with Semaglutide at a 1.0 mg dose resulted in a significant weight reduction (−8.09 kg) and a decrease in HbA1c levels (−1.073%). This significant weight loss was primarily attributed to a lower body mass index (BMI) and weight at baseline, as well as a slightly longer duration of treatment with Semaglutide compared to Liraglutide. These results align with various clinical trials that have examined the effects of Semaglutide at lower doses [23,24,25]. In the STEP 2 trial, Semaglutide at a dose of 2.4 mg administered once weekly was compared to 1.0 mg once weekly in overweight or obese patients with diabetes. The results showed a mean weight change of −7.0% for the 1 mg once weekly Semaglutide dose, alongside an improvement in HbA1c levels of −1.5% [23]. Similarly, the SUSTAIN-6 trial reported a mean body weight reduction of −4.9 kg and a HbA1c decrease of −1.4%, while the SURPASS-2 trial documented a mean HbA1c reduction of −1.86 percentage points, with an average weight loss of −5.7 kg [24].

Furthermore, our findings are consistent with real-world studies that reported an average weight loss of 4.4% and an HbA1c improvement of 0.4% among patients with type 2 diabetes mellitus (T2DM), regardless of their diabetes status [26]. Notably, patients receiving Semaglutide exhibited the highest average age and a greater number of comorbidities; moreover, the prevalence of cardiovascular disease (CVD) among those patients was surprisingly high. Interestingly, the mean number of prescription medications was lower for this group. It is important to acknowledge that the retrospective design of our study may limit the exploration of other potential benefits associated with the use of Semaglutide in real-world settings. Nevertheless, previous clinical trials have established the efficacy of Semaglutide at the 1.0 mg dose for improvements beyond just weight and HbA1c reduction, including enhanced cardiovascular risk outcomes [24]. The data support the ADA’s recommendations regarding the use of glucagon-like peptide-1 receptor agonists (GLP-1 RAs), particularly Semaglutide and Tirzepatide, in managing type 2 diabetes mellitus (T2DM) in individuals with pre-existing cardiovascular disease or those at high risk for cardiovascular disease (CVD). This is due to their proven effectiveness for weight loss, a lower incidence of hypoglycemia, and demonstrable benefits for cardiovascular health [23].

From a financial perspective, our analysis highlights notable differences in the efficacy of Semaglutide at the 1.0 mg dose compared to Liraglutide at the 3.0 mg dose, particularly regarding weight loss, reduction in HbA1c levels, and associated costs. These factors encompass direct medical expenses and medication costs, all of which favor Semaglutide. This finding is consistent with previous studies assessing the cost-effectiveness of Semaglutide 1.0 mg in comparison to other GLP-1 RAs for T2DM [17,18,19].

In terms of comparative outcomes, Semaglutide at the 1.0 mg dose has shown better control over HbA1c levels and greater weight loss in both short- and long-term analyses, leading us to conclude that it represents a cost-effective option when benchmarked against other GLP-1 RAs. For example, a study conducted in Estonia indicated that Semaglutide at a dose of 1.0 mg, administered once weekly, was associated with an improvement in QALY of 0.13 years compared to Liraglutide 1.2 mg. While the direct costs were approximately EUR 67 higher due to increased acquisition prices, the overall cost savings resulting from a reduction in diabetes-related complications made Semaglutide a favorable choice in the context of a cost-effectiveness analysis, yielding an incremental cost-effectiveness ratio of EUR 523/QALY gained, which remains below the willingness-to-pay (WTP) threshold of EUR 52,390 per QALY gained [27].

Further analyses have indicated that among the four evaluated GLP-1 RAs, Semaglutide presents a cost-effective strategy, with an incremental cost-effectiveness ratio (ICER) of USD 135,467 per QALY based on a WTP threshold of USD 195,000 per QALY [18]. These findings are in line with our results; however, our study’s results not only confirmed the greater efficacy of the Semaglutide 1.0 mg dose QW dosage versus the Liraglutide 3.0 mg/day dose for managing elevated HbA1C levels and obesity, but they also demonstrated that its use could lead to significant cost savings.

In conclusion, although Semaglutide is approved at a dosage of 2.4 mg for the management of obesity, irrespective of the presence of T2DM, our findings suggest that the off-label use of 1.0 mg may achieve significant reductions in both body weight and HbA1c levels when compared to the 3.0 mg dosage of Liraglutide, which is specifically formulated for obesity treatment. While the Semaglutide 1.0 mg dose does not have official FDA approval for obesity management, the evidence presented indicates that it could serve as an effective adjunct for obese, prediabetic patients aiming to control their condition. This potential role in obesity management warrants serious consideration.

### Study Limitations

This study utilized real-world data to assess the clinical and financial implications of Semaglutide and Liraglutide from the perspective of public healthcare payers in Saudi Arabia. However, several limitations must be acknowledged. The study used a retrospective single-center design, which comes with inherent limitations, particularly its small sample size. This small sample could significantly impact the generalizability of the findings, making it challenging to apply the results broadly to other healthcare institutions across Saudi Arabia. Although there are notable similarities in patient characteristics—such as demographics, health conditions, and treatment protocols—among various public healthcare facilities within the kingdom, the unique context and specific patient population of a single-center design may not fully capture the diversity and variability found in other institutions. Therefore, it is essential to exercise caution when attempting to extrapolate these findings to different healthcare settings in the region. Additionally, the retrospective nature of the study introduces potential confounding factors, such as medication intolerance, variations in physical activity, and the use of other medications from different hospitals, that were not accounted for. It is important to note that combining physical exercise with GLP-1 receptor agonists (GLP-1RAs) has been shown to yield greater weight reduction and improved glycemic control [28]. However, this significant factor was not controlled for due to the study’s retrospective design, as previously mentioned. Consequently, a larger study with a control group is necessary to address these confounding factors, particularly regarding physical activity levels.

Moreover, there were gaps in the data concerning critical parameters such as HbA1c, weight, and BMI at baseline and during the follow-up period, which may have led to an underestimation of the clinical effectiveness or an overestimation of the costs associated with these medications. Furthermore, issues related to the availability and supply of these medications during the study period impacted patient adherence. Therefore, future research is essential to address the limitations outlined above.

## 5. Conclusions

The findings of this study indicate that administering Semaglutide at a dosage of 1.0 mg once weekly results in a significantly greater reduction in both body weight and HbA1c levels compared to Liraglutide when administered daily at a dosage of 3.0 mg. Specifically, Semaglutide not only facilitates more effective weight loss but also achieves superior glycemic control, as evidenced by the HbA1c metrics. In addition to these health benefits, our findings emphasize that the average annual costs associated with GLP-1 receptor agonists, particularly Semaglutide, are notably lower, leading to reduced overall medication expenses for patients. In contrast, while Liraglutide also provides a meaningful decrease in body weight and HbA1c levels, it comes with higher overall annual medication costs, which may affect its long-term accessibility for patients managing obesity. Therefore, our study highlights the advantages of Semaglutide in terms of both clinical efficacy and economic considerations.

## Figures and Tables

**Table 1:** Patient baseline characteristics.

Characteristic	Liraglutide (n = 100)	Semaglutide (n = 100)	*p*-Value	Total
Age, mean ± SD	43.87 ± 10.87	53.20 ± 11.60	<0.0001	48.54 ± 12.15
Gender, N (%)				
Male	30 (30.0)	39 (39.0)	0.1807	69 (34.50)
Female	70 (70.0)	61 (61.0)		131 (65.50)
Duration of treatment (months), mean ± SD	19.13 ± 5.77	22.01 ± 7.59	0.0029	20.57 ± 6.88
Weight in kg, mean ± SD	99.70 ± 16.18	95.65 ± 18.75	0.1040	97.68 ± 17.59
Body Mass Index (BMI)	38.45 ± 5.23	36.73 ± 5.79	0.0286	37.59 ± 5.57
HbA1c (%)	5.92 ± 0.20	9.00 ± 1.83	<0.0001	7.46 ± 2.02
Comorbidities, N (%)				
Diabetes	0 (0)	100 (100)	<0.0001	100 (50)
Dyslipidemia	16 (16.0)	46 (46.0)	<0.0001	59 (29.5)
Hypertension	19 (19.0)	53 (53.0)	<0.0001	72 (36.0)
Asthma	11 (11.0)	5 (5.0)	0.1179	16 (8.0)
Hypothyroidism	12 (12.12)	10 (10.0)	0.6333	22 (11.06)
Osteoporosis	1 (1.0)	1 (1.0)	1.0000	2 (1.0)
Osteoarthritis	6 (6.0)	1 (1.0)	0.1184	7 (3.50)
Irritable Bowel Syndrome	8 (8.0)	1 (1.0)	0.0349	9 (4.50)
Depression	3 (3.0)	1 (1.0)	0.6212	0.6212
Benign Prostate Hyperplasia	0 (0.0)	1 (1.0)	1.0000	1 (0.50)
Hepatitis B Virus	0 (0.0)	1 (1.0)	1.0000	1 (0.50)
Generalized Anxiety Disorder	9 (9.0)	0 (0.0)	0.0032	9 (4.50)
Migraine	0 (0.0)	1 (1.0)	1.0000	1 (0.5)
Chronic lower back pain	8 (8.0)	2 (2.0)	0.0516	10 (5.0)
Chronic kidney disease	0 (0.0)	1 (1.00)	1.00	1 (0.50)
Iron deficiency anemia	6 (6.0)	2 (2.0)	0.2790	8 (4.0)
Dermatological disorders (e.g., psoriasis, atopic dermatitis)	4 (4.0)	2 (2.0)	0.6827	6 (3.0)
Rheumatoid arthritis	3 (3.0)	0 (0.0)	0.2462	3 (1.50)
Number of comorbidities	1.79 ± 1.5204	2.28 ± 1.07	0.0092	2.04 ± 1.33
Number of prescription medications	5.96 ± 4.51	4.51 ± 1.7203	0.0032	5.24 ± 3.48

**Table 2:** Difference in weight (kg) and HbA1c (%) between baseline and follow-up.

Variable	Mean at Baseline ± SD	Mean at Follow-Up ± SD	Mean Difference (95% Confidence Interval)	*p*-Value
Weight difference (kg)				
Liraglutide	99.70 ± 16.18	93.82 ± 15.81	−5.884 (−7.85 to −3.92)	<0.0001
Semaglutide	95.66 ± 18.75	87.56 ± 15.98	−8.09 (−9.99 to −6.20)	<0.0001
HbA1c difference (%)				
Liraglutide	5.92 ± 0.20	5.62 ± 0.88	−0.298 (−0.47 to −0.126)	0.0009
Semaglutide	9.01 ± 1.83	7.93 ± 1.82	−1.073 (−1.45 to −0.69)	<0.0001

**Table 3:** The mean weight (kg) reduction and direct medical costs for patients on Liraglutide (N = 100) versus Semaglutide (N = 100).

Variable	Semaglutide	Liraglutide	Mean Difference (95% Confidence Interval)
Cost of treatment (USD), mean ± SD	4523.16 ± 2176.04	5787.92 ± 4606.97	−1264.76 (−1826.82 to 33.76)
Difference in weight (kg), mean ± SD	−8.10 ± 9.55	−5.88 ± 9.89	−2.22 (−7.68 to −2.784)

**Table 4:** The mean HbA1c (%) reduction and direct medical costs for patients on Liraglutide (N = 100) versus Semaglutide (N = 100).

Variable	Semaglutide	Liraglutide	Mean Difference (95% Confidence Interval)
Cost of treatment (USD), mean ± SD	4523.16 ± 2176.04	5787.92 ± 4606.97	−1264.76 (−2368.16–−239.686)
Difference in HbA1c (%), mean ± SD	−1.07 ± 1.91	−0.30 ± 0.87	−0.77 (−0.923–−0.0971)

## Data Availability

The data are available upon reasonable request from the corresponding author.
